# Investigation on distribution of airborne fungi in outdoor environment in Tehran, Iran

**DOI:** 10.1186/2052-336X-12-54

**Published:** 2014-03-03

**Authors:** Masoomeh Shams-Ghahfarokhi, Sanaz Aghaei-Gharehbolagh, Narges Aslani, Mehdi Razzaghi-Abyaneh

**Affiliations:** 1 Department of Mycology, Faculty of Medical Sciences, Tarbiat Modares University, 14115-331 Tehran, Iran; 2 Department of Mycology, Pasteur Institute of Iran, 13164 Tehran, Iran

**Keywords:** Airborne fungi, Outdoor air, Hyphomycetes, Zygomycetes, *Aspergillus*, *Cladosporium*, *Penicillium*, *Alternaria*, Tehran

## Abstract

**Background:**

Airborne fungi are responsible for the majority of fungal infections in humans and animals. Outdoor air markedly influences the prevalence of fungal spore levels in indoor air and thus, it is the major source of fungal infections in indoor environments especially in hospitalized individuals.

**Methods:**

Using a settle plate method, air sampling (1092 air samples from 93 sampling sites in 22 geographic regions of Tehran) was performed by exposing 90 mm settle plates containing Malt extract agar and Potato dextrose agar to the air for 30 min. The plates were incubated at 28°C for 2–3 weeks and examined daily for visible fungal growth. Purified fungal colonies were identified at the genus level based on morphological criteria according to standard methods.

**Results:**

A total of 6455 colonies belonging to 24 different fungal genera were isolated. Area V situated in the city center was the most contaminated region with 2523 fungal colonies (39.1%), while area IV in the West showed the least contamination rate (636 colonies; 9.8%). Airborne fungi isolated were classified into 4 classes including hyaline Hyphomycetes (53.5%), dematiaceous Hyphomycetes (41.6%), Zygomycetes (2.8%) and Coelomycetes (0.2%). *Aspergillus* (31.3%) was the most prominent isolated fungus followed by *Cladosporium* (22.1%), *Penicillium* (13.8%) and *Alternaria* (12.2%).

**Conclusion:**

Our results indicate that outdoor air is a potential threat to public health because of harboring a wide array of pathogenic and allergenic airborne fungal spores which can serve as the main source of contamination of indoor environments such as homes, offices and hospitals.

## Introduction

Exposure to airborne pathogens is a major risk factor for human health [1]. It has been shown that microorganisms (e.g., fungi, viruses, and bacteria) from environmental sources may disperse over great distances by air currents and ultimately be inhaled, ingested, or come into contact with individuals who have had no contact with the infectious source. Air pollution from dampness and moulds, chemicals and other biological agents is one of the most public health problems with increasing importance due to the adverse health effects on humans, animals and plants [1-3]. Fungi are an important part of airborne microflora that beyond the air, have been widely distributed in soil, water, and decaying vegetation [4-7]. The importance of airborne fungal contaminants has been dramatically increased in view of health hazards caused by the spores themselves or by microbial metabolites. In addition to the risk for fungal infections from superficial to life-threatening nosocomial types, the allergenic and toxigenic properties, as well as the inflammatory effects may consider as possible health impacts of fungal bioaerosols [8-13]. As an interesting event, it has been shown that fungal spores can harbor considerable amounts of fungal toxic secondary metabolites such as mycotoxins which are toxic when they enter to the host body by inhalation from airborne dust and bioaerosols [14]. Likewise, fungal volatile organic compounds concentrated in outdoor atmosphere have been suggested to affect human health, causing lethargy, headache, and irritation of the eyes and mucous membranes of the nose and throat [14-16].

Among the approximately 100,000 known species of fungi, those of interest in outdoor and indoor environments belong to the class of Deuteromycetes or Fungi Imperfecti, with a few exceptions (e.g. Mucorales, Ascomycetes, wood-rotting Basidiomycetes and some yeasts). Although indoor environment itself is considered as a source of indoor fungi by growing them in building materials, foodstuffs, flower pots, pet bedding materials and house dust, outdoor sources are usually dominant for indoor contamination by pathogenic fungal species [14]. In suitable conditions, filamentous fungi grow and sporulate in various substrates and constitute significant sources of airborne fungal conidia and hyphal fragments in indoor environments. Most outbreaks of nosocomial fungal disease have been attributed to airborne fungi from sources outside of the hospital [17-23]. The typical fungal genera investigated are *Cladosporium*, *Alternaria*, *Aspergillus* and *Penicillium*, probably because they are very often the most prevalent genera in ambient air [24-26]. With respect to the adverse effects on the human health, many studies have been carried out about the fungal community both in outdoor and indoor environments. The genera *Cladosporium*, *Alternaria*, *Penicillium*, and *Aspergillus* comprise the major part of fungal community in the atmosphere. It has been demonstrated that the abundance of airborne fungal spores in outdoor air vary from place to place and it influences mainly by climatic conditions and human activities [1].

Several studies from various parts of Iran such as Sari, Shiraz and so on demonstrated a wide array of microfungi in air samples from outdoor environments [9,27-29]. It can be considered as an important factor in human health and economics. Regarding to the very limited information about diversity and distribution patterns of airborne fungi in Tehran, the capital of Iran as an important factor in public health and economics, the present study was to investigate the diversity and distribution patterns of airborne mycoflora of outdoor environments throughout the city. A total of 1092 air samples were prepared from 93 sampling sites scattered in 22 distinct regions and analyzed for airborne culturable fungal spores.

## Materials and methods

### Sampling sites

**Figure 1:**
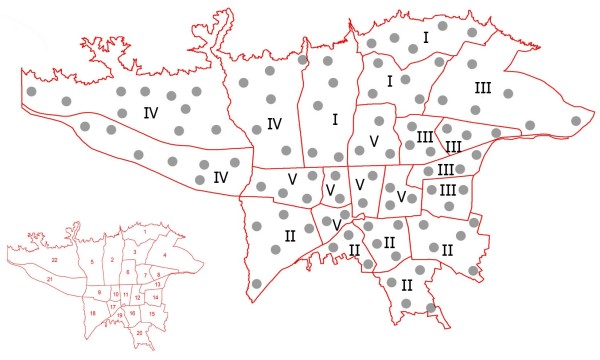
**Tehran map showing 22 distinct geographic regions (small picture) and all 93 sampling sites marked with gray circles (large picture).** Air sampling (1092 samples) was carried out in regions divided into 5 areas including I (locations 1, 2 and 3 with 15 sampling sites and 165 air samples), II (locations 15, 16, 18, 19 and 20 with 21 sampling sites and 231 air samples), III (locations 4, 7, 8, 13 and 14 with 18 sampling sites and 216 air samples), IV (locations 5, 21, 22 with 23 sampling sites and 288 air samples) and V (locations 6, 9, 10, 11, 12 and 17 with 16 sampling sites and 192 air samples).

Tehran is situated at an altitude of 1100–1800 meters above sea level and within the latitude and longitude of 35° 44' N, 51° 33' E. The city is located at the folded zone of Alborz mountains which the deposits are mainly a period of limestone and dolomite and has many rivers and springs. The climate is mountainous temperate and semi-arid with average annual rainfall of approximately 500 mm and a mean relative humidity of 60%. On the basis of Tehran’s map, all 22 district regions (1 to 22) were included in our study. As illustrated in Figure 1, these regions were divided into 5 areas including I (locations 1, 2 and 3 with 15 sampling sites and 165 air samples), II (locations 15, 16, 18, 19 and 20 with 21 sampling sites and 231 air samples), III (locations 4, 7, 8, 13 and 14 with 18 sampling sites and 216 air samples), IV (locations 5, 21, 22 with 23 sampling sites and 288 air samples) and V (locations 6, 9, 10, 11, 12 and 17 with 16 sampling sites and 192 air samples).

### Air sampling

A total of 1092 air samples were prepared from 93 sampling sites during July 2012 using settle plate method according to Hoekstra *et al.*[30]. Sampling sites were chosen according to the extents of studied areas covering overall parts of each location. Plastic plates (9 mm Dia.) contained Malt extract agar (MEA; E. Merck, Darmstadt, Germany) and Potato dextrose agar (PDA; E. Merck, Darmstadt, Germany) with lids open were placed in sampling sites at the height of about one meter above the floor, for 30 min [31]. After that, the plates were transferred to the laboratory for fungal isolation and identification.

### Isolation and identification of airborne fungi

The plates were incubated at 28°C for 2–3 weeks for confidence about growing all airborne fungal conidia and examined daily for any visible fungal growth [31]. Fungal colonies grown on PDA or MEA were purified using agar block and Hyphal tipping methods [32]. Fungal isolates were identified at the genus level according to their microscopic and macroscopic morphological criteria [30].

## Results

**Figure 2:**
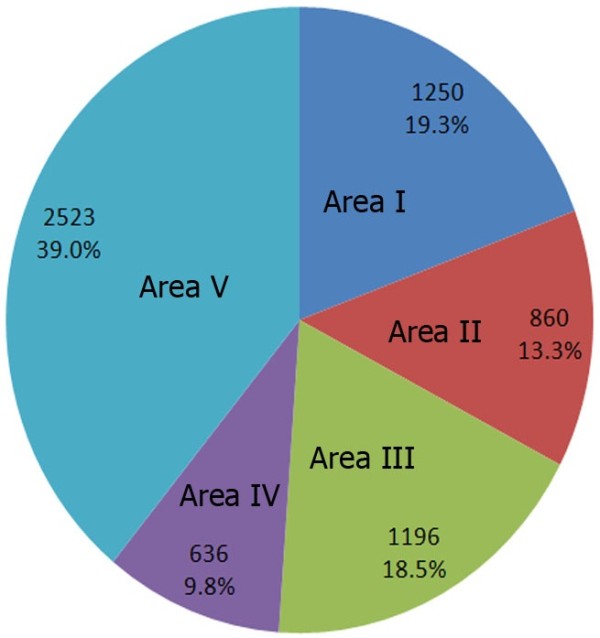
The number of fungal colonies and frequencies of fungal air contamination in five studied areas (I to V) of Tehran is shown.

In the present study, distribution and diversity of outdoor airborne mycoflora in all 22 geographic sections of Tehran was evaluated using settle plate method during summer 2012. A total of 1092 settle plates of outdoor air sampled from 93 sampling sites were studied. As indicated in Table 1, a total of 6455 colonies belonging to 24 different fungal genera (6331 colonies) and sterile mycelia (124 colonies) were isolated. The number of fungal colonies in each region is represented in Figure 2. Area V situated in the city center was the most contaminated region with 2523 fungal colonies (39.1%) followed by areas I in the North (1250 colonies; 19.4%), III in the East (1196 colonies; 18.5%), II in the South (860 colonies; 13.3%) and IV in the West as the least contaminated area with 9.8% contamination (636 colonies). *Aspergillus* (31.3%) was the most prominent fungal genus isolated followed by *Cladosporium* (22.1%), *Penicillium* (13.8%) and *Alternaria* (12.2%). The members of these genera were the only fungi which were isolated from outdoor air of all areas from I to V.

**Table 1:** Frequency and distribution of airborne fungi isolated from 93 sampling sites belonging to 22 distinct regions divided into five major areas of Tehran city, the capital of Iran

**Fungus**	**Area I**		**Area II**		**Area III**		**Area IV**		**Area V**		**Total**	
	**No.**	**%**	**No.**	**%**	**No.**	**%**	**No.**	**%**	**No.**	**%**	**No.**	**%**
*Aspergillus* spp*.*	280	4.3	308	4.8	672	10.4	252	3.9	616	9.5	2128	32.97
*Penicillium* spp*.*	392	6.1	98	1.5	56	0.9	84	1.3	308	4.8	938	14.53
*Cladosporium* spp*.*	196	3.0	294	4.5	154	2.4	99	1.5	756	11.7	1499	23.22
*Alternaria* spp*.*	154	2.4	112	1.7	98	1.5	110	1.7	350	5.4	824	12.76
*Monilia* spp*.*	70	1.1	0	0.0	28	0.4	24	0.4	30	0.5	152	2.35
*Drechslera* spp*.*	28	0.4	14	0.2	0	0.0	12	0.2	55	0.8	109	1.69
*Rhizomucor* spp*.*	0	0.0	0	0.0	14	0.2	15	0.2	84	1.3	113	1.75
*Absidia* spp*.*	14	0.2	0	0.0	0	0.0	0	0.0	0	0.0	14	0.22
*Rhizopus* spp*.*	15	0.2	0	0.0	0	0.0	0	0.0	0	0.0	15	0.23
*Mucor* spp*.*	0	0.0	0	0.0	0	0.0	11	0.2	17	0.2	28	0.43
*Paecilomyces* spp	12	0.2	0	0.0	17	0.3	0	0.0	31	0.5	60	0.93
*Cladophialophora* spp*.*	0	0.0	0	0.0	0	0.0	0	0.0	33	0.5	33	0.51
*Phialophora* spp*.*	0	0.0	0	0.0	30	0.5	0	0.0	0	0.0	30	0.46
*Exophiala* spp*.*	10	0.1	0	0.0	12	0.2	0	0.0	25	0.4	47	0.73
*Phoma* spp*.*	2	0.0	0	0.0	0	0.0	0	0.0	12	0.2	14	0.22
*Epiccocum* spp*.*	0	0.0	0	0.0	0	0.0	0	0.0	12	0.2	12	0.19
*Ulocladium* spp*.*	0	0.0	0	0.0	3	0.0	0	0.0	43	0.7	45	0.70
*Curvularia* spp*.*	0	0.0	5	0.1	0	0.0	0	0.0	28	0.4	33	0.51
*Acremonium* spp*.*	0	0.0	15	0.2	0	0.0	0	0.0	20	0.3	35	0.54
*Fusarium* spp*.*	28	0.4	0	0.0	15	0.2	13	0.2	45	0.7	101	1.56
*Trichothecium* spp*.*	0	0.0	0	0.0	27	0.4	0	0.0	11	0.2	38	0.59
*Stemphylium* spp*.*	0	0.0	0	0.0	18	0.3	0	0.0	0	0.0	18	0.28
*Basidiobolus* spp*.*	0	0.0	0	0.0	0	0.0	0	0.0	11	0.2	11	0.17
*Helminthosporium* spp*.*	26	0.4	0	0.0	0	0.0	6	0.1	0	0.0	34	0.53
Mycelia sterilia	23	0.4	14	0.2	52	0.8	10	0.1	25	0.4	124	1.92
Total	1250	19.4	860	13.3	1196	18.5	636	9.8	2523	39.0	6455	100

**Figure 3:**
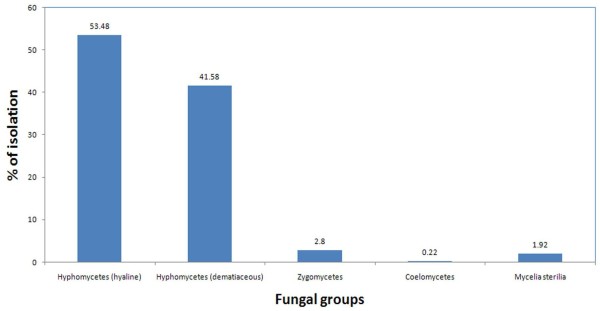
Total frequency of airborne mycoflora distributed in different classes of fungi including Hyphomycetes (hyaline and dematiaceous), Zygomycetes, Coelomycetes and mycelia sterilia.

As shown in Figure 3, airborne fungi isolated were classified into 4 classes including hyaline Hyphomycetes (53.5%), dematiaceous Hyphomycetes (41.6%), Zygomycetes (2.8%) and Coelomycetes (0.2%) as well as mycelia sterilia (1.9%). *Aspergillus* was the most prevalent genus in fungi belonging to hyaline Hyphomycetes, *Cladosporium* in dematiaceous Hyphomycetes and *Rhizomucor* in Zygomycetes. The genus *Phoma* was the exclusive fungus identified in the Coelomycetes with a total frequency of 0.2%.

Besides widely distributed genera of Zygomycetes including *Rhizomucor*, *Mucor*, *Absidia* and *Rhizopus* isolated in the present study from outdoor air, the genus *Basidiobolus* was interestingly reported from eleven samples in Area V account for 0.17% of the total isolates.

## Discussion

In the present study, fungal air quality of outdoor environments of Tehran, the capital of Iran was evaluated by sedimentary sampling using settle plate method. This method is simple and inexpensive and provides an integrated assessment of exposure over longer periods than that of most volumetric methods [14]. All the outdoor air samples prepared from 93 sampling sites of 22 distinct geographic regions were contaminated with different fungi from 24 genera. Majority of isolated fungi were belonging to the hyaline and dematiaceous Hyphomycetes and in a very less extent to Zygomycetes and Coelomycetes. *Aspergillus* was the most predominant fungus accounting for nearly 30% of total isolates followed by *Cladosporium*, *Penicillium* and *Alternaria*. It may be due to the fact that *Aspergillus, Penicillium* and *Cladosporium* produce numerous small and light spores that generally remain in the air for a long period of time, whereas other fungal genera produce fewer, larger and heavier spores which tend to have faster settling [17]. In several surveys of airborne fungal spores, it has been demonstrated that the most prevalent fungi were belonged to the genera *Cladosporium*, *Alternaria*, *Curvularia*, *Aspergillus* and *Penicillium*[1-7,9,13,17-26]. In a study on culturable airborne fungi in outdoor environments in Beijing, China, fourteen fungal genera including 40 species were identified [33]. *Penicillium* was the most abundant species, comprised more than 50% of the total isolates followed by *Cladosporium*, *Alternaria*, *Penicillium* and *Aspergillus*. Wu *et al.*[34] studied ambient fungal aeroallergens in Taipei, Taiwan and reported predominance for *Cladosporium*, *Penicillium*, *Curvularia* and *Aspergillus*. Ataygul *et al.*[35] evaluated the daily distribution of allergic fungal spores in Bursa, Turkey. Ten fungal genera including *Cladosporium*, *Alternaria*, *Aspergillus*, *Penicillium*, *Fusarium*, *Epicoccum*, *Drechslera*, *Pithomyces*, *Stemphylium*, *Chaetomium*, and *Curvularia* were identified with dominancy of *Cladosporium* with 88.11% frequency. Using gravitational settle plate method, occurrence of airborne fungi in the outdoor environment of Trabzon, Turkey was evaluated by Topbas *et al.*[36]. They reported the genera *Penicillium*, *Alternaria* and *Fusarium* as the most prevalent fungi in different seasons. O’Gorman and Fuller [37] studied airborne spore concentrations of selected allergenic and pathogenic fungi in outdoor locations in Dublin, Ireland in 2005. *Cladosporium*, *Penicillium*, *Aspergillus* and *Alternaria* spores were constantly present in the Dublin atmosphere, representing more than 20% of the total culturable spore count. Airborne spores of *A. fumigatus* and basidiospores of *Schizophyllum commune* were detected in some samples. A one-year prospective survey of fungal air contamination was conducted in outdoor air of a French hospital [19]. *Cladosporium* was the dominant genus with a frequency of 55% in total isolated fungi. In a comprehensive study on airborne fungi of outdoor air in Basrah city, southern Iraq, *Cladosporium* was reported as the most dominant species with 31.3% frequency (highest number in winter) followed by *Penicillium notatum* (11.9%), *Alternaria alternata* (10.0%) and *Aspergillus niger* (5.8%) [25].

Several studies have been conducted in Iran and wide arrays of fungi are reported as dominant air mycoflora. In a survey of outdoor air of Qeshm Island, Southern Iran, a predominance of dematiaceous fungi versus hyaline Hyphomycetes were reported by Barrati *et al.*[28]. Theses authors reported that *Alternaria* (63.86%) was the prominent isolated fungus followed by *Cladosporium* (11.81%). Shokri *et al.* reported the genera *Mucor*, *Cladophialophora*, *Alternaria*, *Aspergillus*, *Penicillium*, *Fusarium* and *Rhizopus* as the dominant fungi in the air of forests and seashore areas of Babol city, Northern Iran [29]. In a previous study of airborne mycoflora of outdoor air from hospital environment, we reported the genera *Penicillium*, *Cladosporium*, *Aspergillus* and *Fusarium* had the highest frequency among the total fungi [23]. We did not isolate any yeast-like fungus from Tehran air. This is in accordance with the results of Shokri *et al.* on the air samples of Babol-Iran and in contrast with those reported by Chadeganipour *et al.* indicating 18.6% frequency of yeasts in the air of Isfahan-Iran [27].

It has been reported that more than 180 genera of airborne fungi with worldwide distribution are associated with allergies and serious human and animal infections [7]. As most indoor fungi came primarily from outdoors, it cannot be discarded the impact that an increase in outdoor fungi may have in increasing the risk of fungal diseases in clinical units. Some studies have tried to correlate outdoor fungal concentration and incidence of fungal diseases. The most common species are likely to belong to the genera *Cladosporium*, *Penicillium*, *Alternaria* and *Aspergillus*[1]. Conidia of these fungi have been reported as permanent outdoor air flora throughout the year. Microfungi such as *Cladosporium* spp., *Alternaria* spp., *Epicoccum nigrum*, and *Botrytis cinerea* are known to be an integral part of the fungal air spora outdoors which cover more than 90% of the total fungal spore load. The fungal spore load of penicillia and aspergilli is in the range of 2–10% and 1–3%, respectively [38]. The microfungi of concern in environmental and occupational hygiene are mostly non-pathogenic or facultative pathogenic (opportunistic) species. In the present study, *Alternaria*, *Aspergillus*, *Cladosporium* and *Penicillium* were reported from Tehran air as the most prominent fungi. These fungi are known causative agents of fungal allergies [24]. Besides established role in respiratory allergies, some genera such as the *Penicillium*, *Aspergillus* and *Fusarium* are relevant as mycotoxin producers [39,40]. These fungi are capable of producing a wide range of carcinogenic and life-threatening mycotoxins such as aflatoxins, ochratoxins and trichothecenes with known adverse effects on humans and animal health [14,39]. If airborne fungal spores of these fungi are inhaled down to the bronchia and alveoli, they will be lysed and the human body thereby exposed to primary and secondary metabolites. Inhalation exposure has been suggested to cause acute kidney failure, central nervous system damage and damage of the upper respiratory tract [39,40].

Several species belonging to the genera *Aspergillus*, *Fusarium* and Zygomycetes isolated from outdoor air in the present work are fungal pathogens responsible for infection in immunocompromised patients. Risk patients need to be protected from these fungal pathogens, particularly by isolation in highly-restricted units, as most of these agents are airborne fungi originates from outdoor environment [14].

In general, results of the present study clearly showed that airborne fungi from different groups are present as real contaminants of outdoor air of Tehran. These fungi especially those involved in the etiology of fungal diseases and allergies such as *Aspergillus*, *Penicillium*, *Cladosporium* and *Alternaria* must be considered as major threats for public health because they are main sources of contamination of indoor environments such as homes, offices and hospitals.

## Competing interests

All authors declare that they have no competing interest.

## Authors’ contributions

MSG and MRA participated in the design of the study and MSG supervised the work. SAG and NA did the analyses, and/or interpreted the analyzed results. MSG and MRA wrote the initial draft and revised the paper critically for important intellectual content and compiled the work in accordance to journal format. All authors have read and approved the final manuscript.
